# Comparison of α-glucosyl hesperidin of citrus fruits and epigallocatechin gallate of green tea on the Loss of Rotavirus Infectivity in Cell Culture

**DOI:** 10.3389/fmicb.2015.00359

**Published:** 2015-04-29

**Authors:** Steven M. Lipson, Fatma S. Ozen, Samantha Louis, Laina Karthikeyan

**Affiliations:** ^1^Department of Biology and Health Promotions, St. Francis College, BrooklynNY, USA; ^2^Department of Biology, New York City College of Technology, The City University of New YorkBrooklyn, NY, USA

**Keywords:** rotavirus, infectivity titers, a-glucosyl hesperidin, epigallocatechin gallate, ELISA

## Abstract

A number of secondary plant metabolites (e.g., flavonoids) possess antiviral/antimicrobial activity. Most flavonoids, however, are difficult to study, as they are immiscible in water-based systems. The relatively new semisynthetic α-glucosyl hesperitin (GH), and the natural plant product epigallocatechin gallate (EGCG) are unique among most flavonoids, as these flavonoids are highly soluble. The antiviral activity of these plant metabolites were investigated using the rotavirus as a model enteric virus system. Direct loss of virus structural integrity in cell-free suspension and titration of amplified RTV in host cell cultures was measured by a quantitative enzyme-linked immunosorbent assay (qEIA). After 30 min. 100 × 10^3^ μg/ml GH reduced RTV antigen levels by ca. 90%. The same compound reduced infectivity (replication in cell culture) by a similar order of magnitude 3 to 4 days post inoculation. After 3 days in culture, EGCG concentrations of 80, 160, and 320 μg/ml reduced RTV infectivity titer levels to ca. 50, 20, and 15% of the control, respectively. Loss of RTV infectivity titers occurred following viral treatment by parallel testing of both GH and EGCG, with the latter, markedly more effective. Cytotoxicity testing showed no adverse effects by the phenolic concentrations used in this study. The unique chemical structure of each flavonoid rather than each phenolic’s inherent solubility may be ascribed to those marked differences between each molecule’s antiviral (anti-RTV) effects. The solubility of EGCG and GH obviated our need to use potentially confounding or obfuscating carrier molecules (e.g., methanol, ethanol, DMSO) denoting our use of a pure system environ. Our work further denotes the need to address the unique chemical nature of secondary plant metabolites before any broad generalizations in flavonoid (antiviral) activity may be proposed.

## Introduction

Reports have linked the consumption of selected plant juices (e.g., cranberry, grape) and green tea to numerous broad-based health benefits. Such health-promoting effects have been traced to certain secondary plant metabolites within these plant species, including proanthocyanidins of berries and grapes, and epigallocatechin gallate (EGCG) of green tea (Higdon and Frei, 2003; Aron and Kennedy, 2008; Bose et al., 2008; Xiao et al., 2011). Among those numerous health promoting effects proposed from the consumption of comestible plant products, antimicrobial activity has been the subject of an increasing number of substantive reports over the last one or two decades. Whether on a descriptive or mechanistic approach, *in vitro* and *in vivo* studies have proposed that flavonoids (phenol-based secondary plant metabolites (Thilakarathna and Vasantha Rupasinghe, 2013) impart antimicrobial activity among a wide range of infectious agents including but not necessarily limited to bacteria (viz., *Escherichia coli*-associated urinary tract infections), viruses (e.g., Herpes Simplex Virus, Human Immunodeficiency Virus, Respiratory Syncytial Virus, Hepatitis C virus, Reovirus, Rotavirus, Enteroviruses,), and parasites (*Entamoeba histolytica, Giardia lamblia*; Mukoyama et al., 1991; Nagi et al., 1992; Calzada et al., 2001; Nair et al., 2002; Mim and Hart, 2003; Suzutani et al., 2003; Lu et al., 2004; Zakay-Rones et al., 2004; Song et al., 2005; Mukhtar et al., 2008; Guay, 2009; Kim et al., 2010; Lipson et al., 2010; Gescher et al., 2011; Roh and Jo, 2011; Thapa et al., 2012). Most flavonoids, however, are immiscible in water and therefore, require carrier organic solvents [e.g., methanol, ethanol, dimethylsulfoxide (DMSO)] to bring such secondary plant metabolites into solution. Dilution of solubilized stock flavonoid preparations to working strength concentrations (e.g., in the preparation of dose-response curves) in cell culture maintenance medium (MM), phosphate-buffered saline (PBS), or other water-based systems, induces a solute precipitation and/or one must maintain a high solvent concentration potentially affecting overt or subtle cytotoxic effects (see Lipson et al., 2013). As alluded to by others, the low water solubility of flavonoids in general, has limited the use of such secondary plant metabolites in basic and biomedical research (Yamada et al., 2006; Saha et al., 2009). A need exists to evaluate water soluble flavonoid extracts for the purpose of identifying new comestible plant products with potential antiviral activity. The semisynthetic α-glucosyl hesperidin (GH) or more commonly termed GH (Hijiya and Miyake, 1999), is an extremely soluble semi-synthetic flavanone of citrus fruit species, promoting a solubility in water ca. 10,000 times that of hesperidin *per se* (Yamada et al., 2006; Saha et al., 2009). Although GH was originally formulated for use as a cosmetic to improve skin circulation and tone (Lanzendorfer et al., 1999; Hou et al., 2012), this product was shown to have an inhibitory effect on the *in vitro* replication of influenza virus, expounding upon the chemical’s marked solubility (Saha et al., 2009). The testing and potential use of flavonoids as antiviral moieties from comestible plant species is supported by an absence of data suggesting any mutagenic activity. One should further point out that despite the availability of RTV vaccines, the “take” is not absolute. Species within the Genus Rotavirus (Family Reoviridae) accordingly, remain a significant etiologic agent of morbidity and mortality, especially in the Third World. The purpose of this study therefore was to evaluate the efficacy of semisynthetic flavonoid GH, as an antiviral secondary plant metabolite using the environmentally significant rotavirus as a model system. In complement with this work, the anti-rotavirus activity of GH was compared where appropriate, to the well-studied and characterized EGCG of green tea, through the determination of changes in RTV infectivity titers in African Green monkey kidney epithelial (MA-104) host cell cultures.

## Materials and Methods

### Virus, Cell Culture, and Infectivity Testing

The simian rotavirus strain SA-11 (RTV; ATCC VR-1565) was used in this study. Infectivity titration measurements were performed to quantitate the number of infectious RTV particles present in our system following treatment with GH and/or EGCG. Cell growth and maintenance were performed according to standard procedures (Lipson, 1992). Briefly, host cells consisted of African green monkey kidney epithelial (MA-104) cells, clone *Cercopithecus aethiop* (ATCC CRL-2378.1) were grown to confluency in 96-well flat bottom microtiter plates. Growth medium (GM) consisting of Eagle’s minimal essential medium supplemented with 10% fetal bovine serum (FBS), 100 μg/ml streptomycin, 100 units penicillin, 1% L-glutamine, and 1% amphotericin B. Maintenance medium (MM) was the same as GM except that 2% FBS was used as a medium supplement. Prior to virus inoculation, the GM was removed by aspiration, with MM added to each well.

The infectivity titer of RTV stock was determined by end-point dilution and expressed as tissue culture infective dose-fifty (TCID_50_)/ml (Reed and Muench, 1938). The virus was frozen at -70°C in aliquot preparations until use.

### Secondary Plant Metabolites

The secondary plant metabolites (–)-EGCG of *Camellia sinensis* (“Tea catechin”) was purchased from Cayman Chemical Co., Chicago, IL, USA. α-GH was kindly supplied by Hayashibara Co. Ltd. (Okayama, Japan). Stock concentrations of GH were prepared in Dulbecco’s PBS modified without calcium and without magnesium (Sigma-Aldrich, Saint Louis, MO, USA), and used immediately. For EGCG, 1 ml of the DPBS was added to manufacturer-supplied screw capped glass vials containing 50 mg of the catechin, inverted numerous times to affect placement of the metabolite into solution, used immediately, or placed for 2–3 days at refrigeration temperature (4°C) until use (Zhou et al., 2003). Dilution of the GH and EGCG to working strength concentrations coupled with the presence of antibiotics/antimycotic supplements in the cell culture MM, precluded any subsequent bacterial/fungal contamination.

#### Experimental Protocols

Anti-RTV activity was evaluated by direct specimen testing in cell-free suspension, and by changes in viral infectivity titers in M-104 cells cultures.

##### Direct Specimen Testing

Direct specimen testing was performed to identify the effect of GH on the loss of RTV capsid antigen integrity in *cell-free* suspension. Briefly, equal volumes of virus stock suspension (3.1 × 10^4^ TCID_50_/ml; **Figure 2**) and PBS containing increasing concentrations of GH were incubated for 5 or 30 min. at room temperature (23°C) followed by direct testing for the presence of viral capsid protein. The positive control consisted of the RTV plus and equal volume of PBS. The negative control consisted of PBS, alone. Direct specimen testing (i.e., used in the current work for the testing of viral capsid function and as an antigenic determinant) in *cell-free* suspension was measured through the use of a quantitative antigen capture [capsid protein VP6 (VP6)] enzyme-linked immunosorbent assay [Premier^TM^ Rotaclone^®^ Enzyme-Linked Immunosorbent Assay, qEIA; (Meridian Diagnostics, Cincinnati, OH, USA)]. Quantitative EIA signals, reflective of changes in viral capsid antigen levels and presented on dose response curves, were objectively measured employing a Genesys 20 spectrophotometer (ThermoFisher, Inc., Waltham, MA, USA) at a wavelength of 450 lambda as per manufacturer’s specifications (Package Insert, Meridian). All testing was performed in triplicate. Developed in the 1980s, the rotavirus antigen capture EIA, has since been used as a sensitive and specific system by medical laboratory personnel and infectious disease specialists for the monitoring of viral antigen as well as RTV (Type A) clinical disease, respectively, (Cukor et al., 1984; Stals et al., 1984; Knowlton et al., 1991; Dornai et al., 1993; Raj et al., 2007; Fang et al., 2009; Tran et al., 2010; Lipson et al., 2011; Gautam et al., 2013; Sai et al., 2013; Lipson, Personal observation). The RTV antigen capture EIA, as well as most other antigen/antibody capture EAI systems, may readily be exploited in a quantitative manner as performed in the current study (see EDVOTEK, 2011).

##### Antigen Detection – Cell Culture Amplification

**FIGURE 1:**
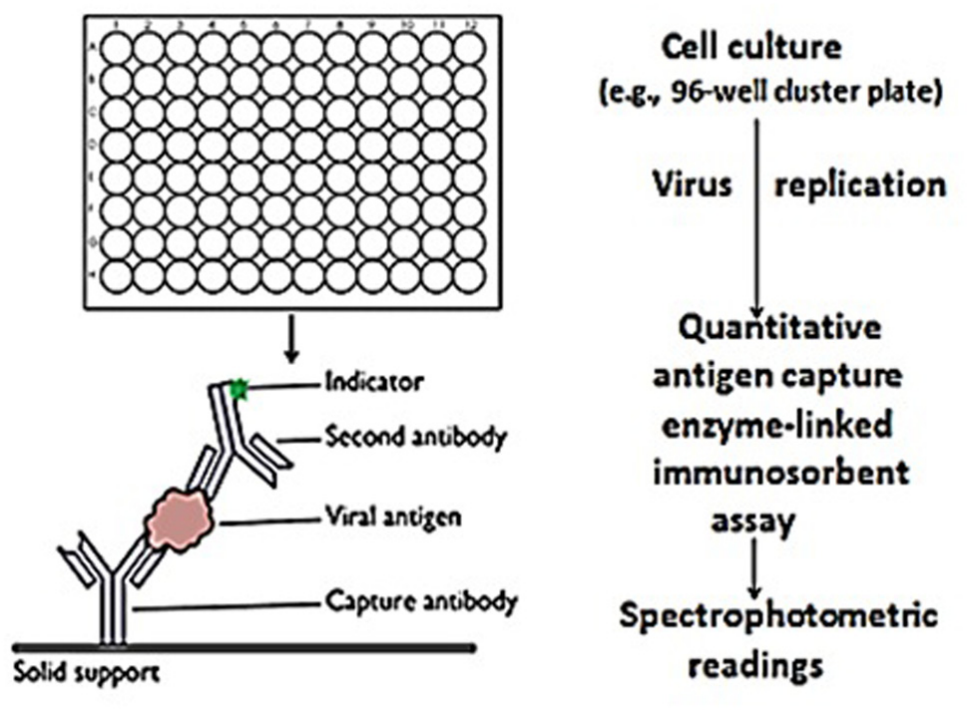
**Rapid *in vitro* determination of rotavirus infectivity.** Rotavirus/flavonoid preparations (and controls) were added in triplicate to MA-104 grown in 96-well cluster plates. After 5 days, the viral capsid protein (i.e., VP6) was quantitatively measured by antigen capture enzyme-linked immunosorbent assay using spectrophotometric readings as the objective signal standard. Spectrophotometric readings were reported as the mean ± SE of the means. *P*-values <0.05 were considered statistically significant.

The technique of antigen detection – *cell culture* amplification (Ag-CCA) technology was used as a methodology to measure changes in viral infectivity titers. Briefly, RTV infectivity titers were performed using MA-104 (host) cells grown to monolayer cultures in 96-well microtiter cluster plates (**Figure 1**). Equal volumes of RTV stock suspensions (3.1 × 10^3^ and 3.1 × 01^4^ TCID_50_/ml and PBS containing 100 × 10^3^ μg/ml GH were inoculated (25 μl) onto 96-well microtiter plates containing MA-104 (host) cells cultures. The cells cultures were incubated for a period of 120 h (5 days) at 37°C in 5% CO_2_. Detection of the presence (or absence) of RTV antigen virus replication as a factor of time, was measured by the qEIA. Objective measurements of changes in capsid antigen concentration/infectivity titers in the host cell cultures (viz., as determined by qEIA signal intensities) were performed spectrophotometrically (Genesys 20 spectrophotometer). Comparative testing of GH and EGCG on the loss of RTV infectivity titers was performed as described above, but the RTV was challenged by varying concentrations of GH (62.5–5,000 μg/ml) and EGCG (0.19–575 μg/ml) suspended PBS. The suspensions were incubated for a periods of 60 min., followed by inoculation of 25 μl of each preparation onto MA-104 cell culture monolayers grown in 96-well flat bottom microtiter plates. The cells cultures were incubated as described above. The supernatants were then tested for changes in infectivity titers using the qEIA (see Lipson et al., 2001a). Positive controls were performed exactly as experimentals, but in the absence of GH or EGCG. All experiments were performed in triplicate.

### Cytotoxicity Testing

Cytotoxicity testing was performed using the Toxilight^R^ Non-Destructive Cytotoxicity Bioassay kit (Cat. No. LT37-619; Lonza, Rockville, MD, USA). Testing was performed in 96-well flat bottom polypropylene cluster plates containing MA-104 cells grown to monolayer culture, as per manufacturer’s specifications. The assay is a quantitative measure of cellular (membrane) damage through the release of adenylate kinase (AK) driving in part, the classic lucinferin/luciferase assay to affect an ATP “spark.” Briefly, increasing concentrations of GH and EGCG were added to MA-104 culture monolayers and incubated for 48-h at room temperature (23°C). Cytotoxicity testing was performed after the incubation period. The positive control consisted of an ammonium chloride-based lysing reagent (Lonza). The negative control consisted of PBS. Readings were reported as relative light units (RLUs) employing a Modulus luminometer (Turner Biosystems/Promega Corp., Sunnyvale, CA, USA).

### Statistics

All experiments were performed in triplicate. Data points are reported as the mean ± SE of the means. *P*-values <0.05 were deemed statistically significant.

## Results and Discussion

The continual occurrence of resistant microorganisms through the selective pressures of antibiotic and antiviral use, remains an important issue in patient care and management. The successful *in vitro* and to a lesser number, *in vivo* application of natural plant products as antiviral moieties, foster the need for continued research with selected plant metabolites as alternative medicines, supplements, and perhaps structural models for the design of new therapeutic agents (Nagi et al., 1992; Nance and Shearer, 2003; Lipson et al., 2010; Jin, 2013).

Alpha-GH, a semisynthetic flavanone extracted from citrus fruits, was originally manufactured as a cosmetic to improve skin tone and circulation. More recently, GH was shown effective as an antiviral, through the blockage of influenza virus replication by the inhibition of viral sialidase needed for viral penetration and egress (Saha et al., 2009). These findings, coupled with the product’s extremely high solubility, prompted us to address this agent’s efficacy against an unrelated infectious virus – the RTV. Comparative testing using the well-studied and characterized catechin, EGCG of the evergreen plant *C. sinensis* (viz., used in the manufacture green tea), was performed as well (Steinmann et al., 2013).

**FIGURE 2:**
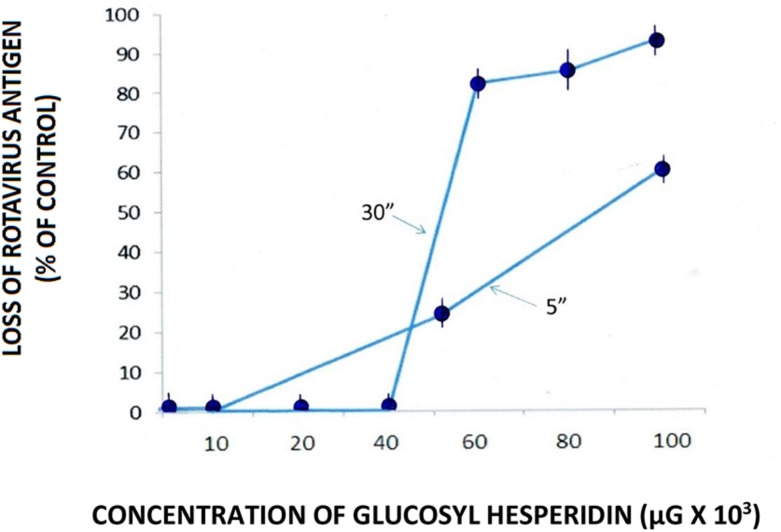
**Effect of glucosyl hesperidin (GH) on the loss of rotavirus (RTV) antigen in cell-free suspension.** RTV/GH complexes were maintained for 5 and 30 min. at room temperature (23°C) followed by the quantitative measurement (using qEIA) of the VP6 capsid antigen. Data points from spectrophotometric readings represent the mean ± SE of the means. *P* < 0.05 is assumed statistically significant.

Direct viral testing of RTV by GH in cell-free suspension using the qEIA showed a loss of viral capsid antigen/integrity starting at a GH input concentration of 50–60 × 10^3^ μg/ml. An increased loss of viral antigen occurred after an incubation period of 30 rather than 5 min., indicating a temporally associated effect by the semi-synthetic flavanone upon the viral capsid protein (**Figure 2**). The loss of viral antigen by GH more specifically, suggests an effect by the semisynthetic flavonoid on the integrity, or at the very least, the availability of VP6 antigen, most probably denoting an effect by the flavonoid on the virion’s (inner) capsid protein. It would not be unreasonable to suggest a flavonoid mediated direct affect on RTV VP6 capsid protein and in turn, a blockage of the particle’s antigenic determinants’ binding to the EIA capture antibody. Supportive to this suggestion is our recent work using transmission electron microscopy, showing an enshrouding or total entrapment of RTV by soluble and particulate proanthocyanidins (Lipson et al., 2011, 2012). As similarly recognized among the proanthocyanidin group of flavonoids, hesperidin and its aglycone hesperitin function in part, as protein adsorbents, supporting our proposed mechanism of GH-associated RTV capsid antigen loss of activity (see the works by Ding et al., 2012; Chakraborty et al., 2013; Shen and Lu, 2013). On the organismic level notably, it is interesting to note that the water soluble trisaccharide GH retains the same biological activities as its rutinose-contaning hesperidin glycoside core molecule (Yamada et al., 2006).

Application of the cell-free assay system as performed herein, although indicative of the semisynthetic flavonoid’s effect on VP6 binding to its complementary antibody, limits our interpretation of these findings to that which is taking place at the flavonoid-virus interface. Antigen detection (e.g., useful in the identification of protein transport, binding, etc.) is indeed important in viral studies. However, and in complement to our use of the qEIA to detect RTV capsid antigen binding in *cell-free* suspension *per se*, comparative infectivity titer determination was performed to place a substantive argument for the potential application (or lack thereof) of GH as an antivirus (anti-RTV) moiety on the cellular level. Accordingly, testing was performed to determine the effect of GH on the “growth” (i.e., changes of infectivity titer over time) of RTV in MA-104 host cells. Further comparative testing using the well-studied and characterized green tea extract EGCG of *Camellia senensis* where appropriate, was evaluated on the cellular level as well. The latter effort was achieved using Ag-CCA, originally developed parenthetically, to fill the void for a rapid viral diagnostic technique in the biomedical setting. The basic concept of Ag-CCA as an infectivity titration assay (viz., the replication of virus in host cell cultures followed by confirmatory/quantitative viral antigen detection (**Figure 1**), has been well described and extensively evaluated employing such techniques as end point titration [tissue culture infectious dose -fifty (TCID_50_), immunofluorescence, immunoperoxidase, polymerase chain, and other substantive assay systems (Vacquier and Cardiff, 1979; Michalski et al., 1986; Swenson and Kaplan, 1987; Cowley et al., 1992; Lipson et al., 2001b; Perez-Ruiz et al., 2003; Terletskaia-Ladwig et al., 2008).

**FIGURE 3:**
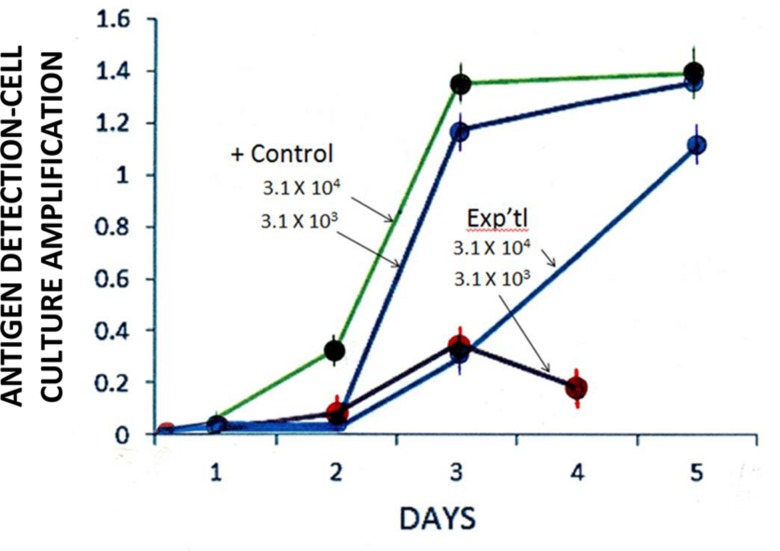
**Effect of GH (100 × 10^3^ μg/ml) on the isolation [antigen detection-cell culture amplification (Ag-CCA)] of RTV in MA-104 cell culture monolayers.** Input titers: 3.1 × 10^3^ to 3.1 × 10^4^ TCID_50_/ml. Day 2 experimental (3.1 × 10^4^) vs. Positive control (3.1 × 10^4^ TCID_50_/ml): *p* = 0.08 (*n* = 3). Spectrophotometric readings performed at 450 nm. Data points represent the mean ± SE of the mean. *P* < 0.05 was assumed statistically significant.

Among GH-treated RTV particles, infectivity titration testing showed a lag in viral growth some 24–48 h post inoculation (p.i.; **Figure 3**). These observations may simply be ascribed to a viral eclipse (Shors, 2009). After 72 and 96-h p.i., however, significant differences in infectivity titers between experimental and control systems were recognized. One-hundred × 10^3^ μg/ml GH affected a loss of RTV infectivity to approximately 75 and 85% of control at 3 and 4 days p.i., respectively, with GH appearing slightly less effective at the lower viral input concentration (viz., 3.1 × 10^3^ vs. 3.1 × 10^4^ TCID_50_/ml; **Figure 3**). Such differences in infectivity titers, i.e., as measured by viral growth (or more appropriately termed, viral replication), may be explained by those differences in the experiment’s input viral concentrations. In accord with that defined by the Poisson distribution, an increase in viral input concentration, viz., the ratio of viral particles to the host cell population or multiplicity of infection would reflect an increase the number of cells infected in a given assay system (Racaniello, 2011). Viral yield accordingly, would be reflected by respective changes in infectivity titers over time.

Anti-RTV activity appeared to be reduced some 120-h (5 days) p.i. (**Figure 3**), suggesting that GH inhibitory activity wanes with time. This suggestion is supported by the known stability (or instability) of GH, whereby the semisynthetic flavanone in question readily hydrolyzes to its insoluble aglycone state (Hijiya and Miyake, 1999); One may not rule out a change in GH solubility thereby reducing the molecule’s dispersion in cell culture and in turn, a reduction in the flavanone’s effect.

Several commercially available secondary plant metabolites showed antiviral activity to the wild type RTV Wa (human) strain in MA-104 cells cultures. The flavanone hesperidin and the flavone diosmin (a semsynthetic modified hesperidin) displayed the greatest RTV inhibitory activity at inhibitory concentration-fifty (IC_50_) endpoints of 10 μM (ca., 0.006 μg/ml); IC_90_ or greater inhibitory values were not determined (Bae et al., 2000). The antiviral effect was ascribed to the rutinose (6-*O*-α-L-rhamnosyl-D-glucose) “R” group moiety of each flavonoid in question, with monolayer pre-treatment showing the greatest inhibitory effect. The anti-RTV effect shown by Kim and co-workers (Bae et al., 2000) however, may not be considered indicative of a hesperidin-associated broad-scale antiviral activity, as suggested by a paucity of such hesperidin-virus studies within the last 10 or more years. The extremely low solubility of hesperidin in water (viz., <0.01%) and the need to use obfuscating organic carrier molecules to bring the hesperidin molecule into solution, has been suggested to be a limiting factor in the use of this flavonoid in antiviral research (Saha et al., 2009). Paredes et al. (2003) notwithstanding, did address the potential antiviral activity of hesperidin as well as the related naringin (glycosides of the aglycones hesperitin and naringenin), showing no inhibitory activity on a Sindbis virus neurovirulent strain used as a test system. The recent work by Lipson et al. (2013) albeit through quantitative viral antigen testing, showed no direct effect by hesperidin, diosmin, nor the flavonone naringin, on the RTV strain SA-11. In the current study, use of the highly soluble GH interestingly, necessitated inordinately high concentrations to approach that antiviral activity shown by Bae et al. (2000) using the naturally occurring (rutinose-containing) hesperidin plant metabolite. These findings, perhaps initially viewed as contradictory, may actually be ascribed to small variations (viz., disaccharide vs. trisaccharide “R” groups on the hesperidin molecule) and/or the recognition of viral strain differences (viz., the human Wa vs. the primate RTV SA-11 strains) between RTV particles under investigation. Similarly, small structural changes between numerous flavonoid types has been recently shown to significantly affect the extent of antiviral (canine distemper virus) activity in Vero host cell cultures (Carvalho et al., 2013). It is also interesting to note that the marked solubility of GH apparently had no significant effect on reducing the semisynthetic flavanone’s high concentrations necessary to invoke an anti-RTV response in the virion’s host cell.

Broad generalities [except perhaps with EGCG (see below) and to a lesser extent, the proanthocyanidins] in flavonoid antiviral studies must be made with caution. As seen in the works of others and in the current investigation specifically, variables including differences in sugar structures, viz., the disaccharide rutinose “R” group of hesperidin vs. the trisaccharide “R” group on GH (Bae et al., 2000; Saha et al., 2009; Anonymous, 2010; Schubert et al., 2010), differences in the type of carrier molecule (e.g., DMSO, methanol, ethanol) necessary to bring immiscible flavonoids into solution (Paredes et al., 2003; Lipson et al., 2013), and the use of different virus groups or strains utilized in a given experimental system (Su et al., 2010; Jin, 2013), make it difficult to generalize on the antiviral efficacy of most flavonoid types.

Health promoting activities by EGCG, the major catechin of green tea, places this molecule in the forefront of flavonoid research. EGCG indeed remains one of the most studied natural plant products of the last one or two decades (Jin, 2013). Green tea consumption and the experimental use of EGCG both *in vitro* and in the animal model, have shown these products to possess anti-cancer, anti-obesity, anti-atherosclerotic, anti-diabetic, as well as anti-microbial activity (Suzuki et al., 2012). EGCG has also been shown to possess marked broad scale antiviral activity as well, among such unrelated viral species/groups as the Human immunodeficiency virus type I, the Human T-cell lymphotrophic virus type I, Hepatitis B and C viruses, Herpes simplex virus types 1 and 2, the Epstein-Barr virus, Human Papilloma virus, Influenza virus, rotavirus, and several enterovirus strains (Mukoyama et al., 1991; Yamaguchi et al., 2002; Bharti et al., 2009; Jin, 2013; Lipson et al., 2013; Steinmann et al., 2013). These findings prompted us to evaluate in parallel, the broadly effective EGCG green tea extract with the relatively new GH. The purpose of such testing was dual, not only to simultaneously compare rotavirus loss of infectivity by each plant extract/semisynthetic product in cell culture, but to re-confirm the efficacy of our quantitative Ag-CCA infectivity titer assay as performed in the current research setting.

Loss of RTV infectivity titers in host cell cultures was minimal 3 days p.i. following inoculation of monolayers with GH/RTV solutions at GH concentrations ranging from 0.063 to 5 mg/ml.

**FIGURE 4:**
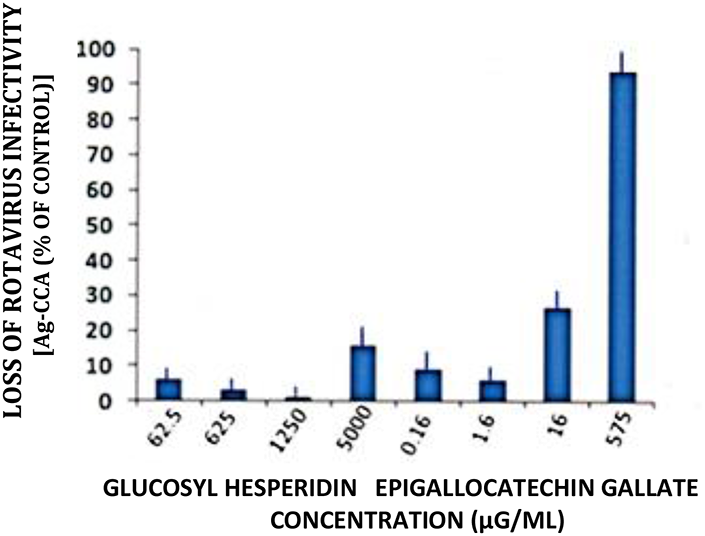
**Loss of rotavirus (RTV) infectivity following treatment with glucosyl hesperidin and epigallocatechin gallate (EGCG).** A markedly reduced concentration of EGCG was effective in reducing RTV infectivity by 1 log_10_. Virus treatment with glucosyl hesperidin failed to approach that of EGCG, regardless of using the inordinately high flavanone concentration of 5000 μg/ml. Mean ± SE of the mean. *P* < 0.05 was assumed statistically significant.

However, under identical treatment conditions, but using 0.58 mg/ml, EGCG reduced RTV infectivity titers by greater than one order of magnitude (i.e., > 90%; **Figure 4**). These findings not only denote the validity of our infectivity titration assay in a comparative evaluation setting, but again point out the relative superiority of EGCG as an antiviral plant product.

**FIGURE 5:**
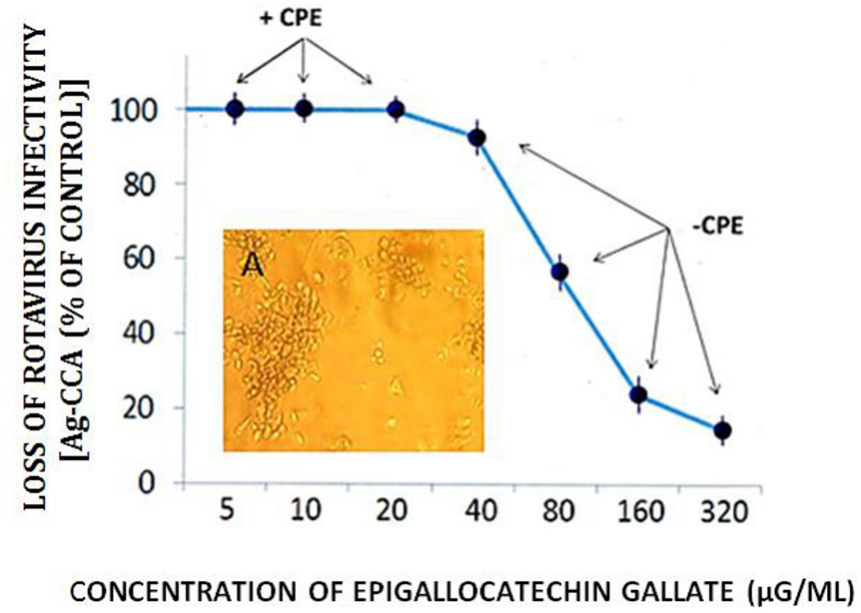
**Effect of EGCG on RTV infectivity in MA-104 host cell cultures.** RTV was treated with increasing concentrations of EGCG, incubated in suspension for 60 min., followed by inoculation of 25 μl of the virus-catechin complex into microtiter plates (96-well cluster plates) containing MA-104 cells in monolayer culture. After 5 days in culture, viral capsid antigen levels were quantitatively determined. The appearance (or absence) of the cytopathic effect (CPE) was recorded as well. Replicate cultures were monitored for a period of 8 days, showing no differences in the appearance of the RTV CPE. Data points based on spectrophotometric readings represent the mean ± SE of the means. *P* < 0.05 was assumed statistically significant. 160 vs. 320 μg/ml: *P* = 0.24. Insert A Photomicrograph of the RTV CPE shows a breakage of cell monolayer with the appearance of cellular rounding and clumping (see Lipson, 1992). Mock infected cell cultures remained intact for the duration of the experiment (not shown).

**FIGURE 6:**
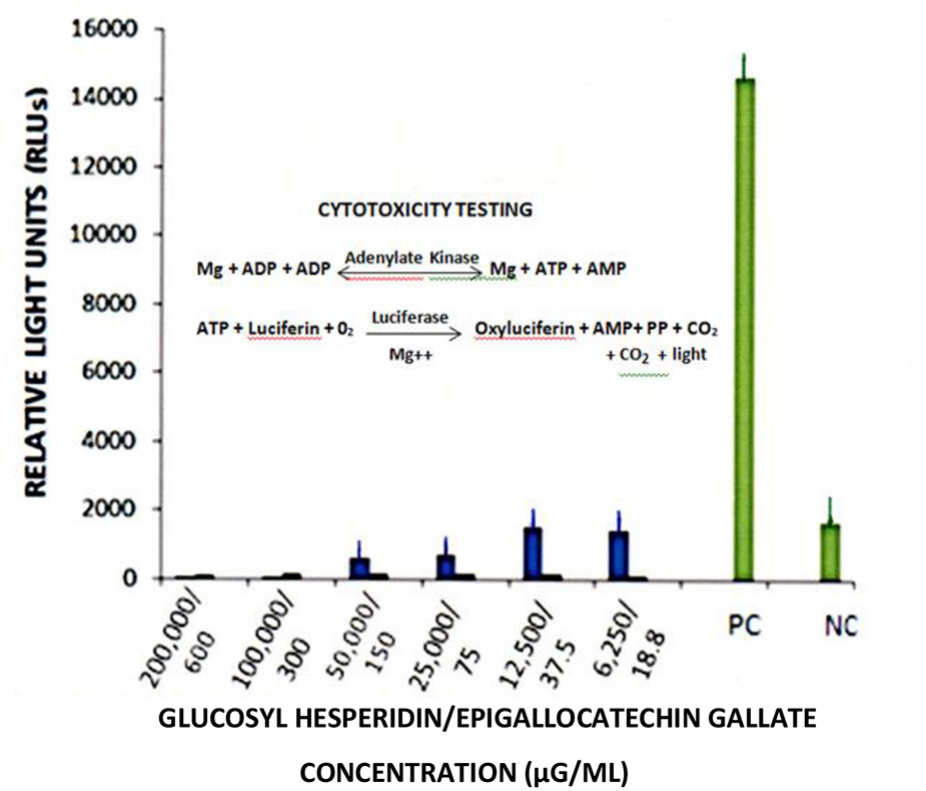
**Cytotoxicity testing of glucosyl hesperidin and epigallogatechin gallate MA-104 cell cultures.** Cytotoxicity testing employed the “Toxilight^R^ Non-Destructive Cytotoxicity Assay”. Readings were performed using a Modulus luminometer (Turner Biosystems/Promega Corp., Sunnyvale, CA, USA) with data presented as relative light units (RLUs). Data points represent the mean ± SE of the means. *P* < 0.05 were assumed statistically significant.

Dose response curves showed a loss of RTV infectivity titer by EGCG to approximately 70 to 80% at concentrations ranging from 160 to 320 μg/ml; No significant differences in anti-RTV activity between 160 and 320 μg EGCG was recognized (*P* = 0.24). A CPE was not recognized by treatment of monolayer cultures with EGCG concentrations >40 μg/ml (**Figure 5**). These values approximated our earlier findings of VP6 loss of activity in cell-free suspension (Lipson et al., 2013). As shown in the current work and that presented by others, EGCG remains a highly effective antiviral natural plant product. The EGCG not only displays consistent antiviral activity among numerous unrelated viral genera or groups, but achieves this effect at reduced concentrations. For example, concentrations ranging from 23 to 46 μM (10.5 to 21 μg EGCG/ml ) readily inhibit infectivity titers from>1 to 3 log_10_ (>90–99.9%) among such unrelated infectious agents as herpes simplex virus, hepatitis C virus, or enterovirus 71 (Isaacs et al., 2008; Ho et al., 2009; Calland et al., 2012). We wish to point out that inaccurate or obfuscating RTV infectivity titer levels due to possible GH- or EGCG-associated cytotoxicity, was non-existent in the current work (**Figure 6**). GH and EGCG at concentrations ranging from 6.2 to 200 mg/ml and 0.18 to 0.6 mg/ml, respectively, (ranges encompassing that used in the present study), failed to produce any gross cell morphologic changes or alterations of host cell membrane integrity.

**FIGURE 7:**
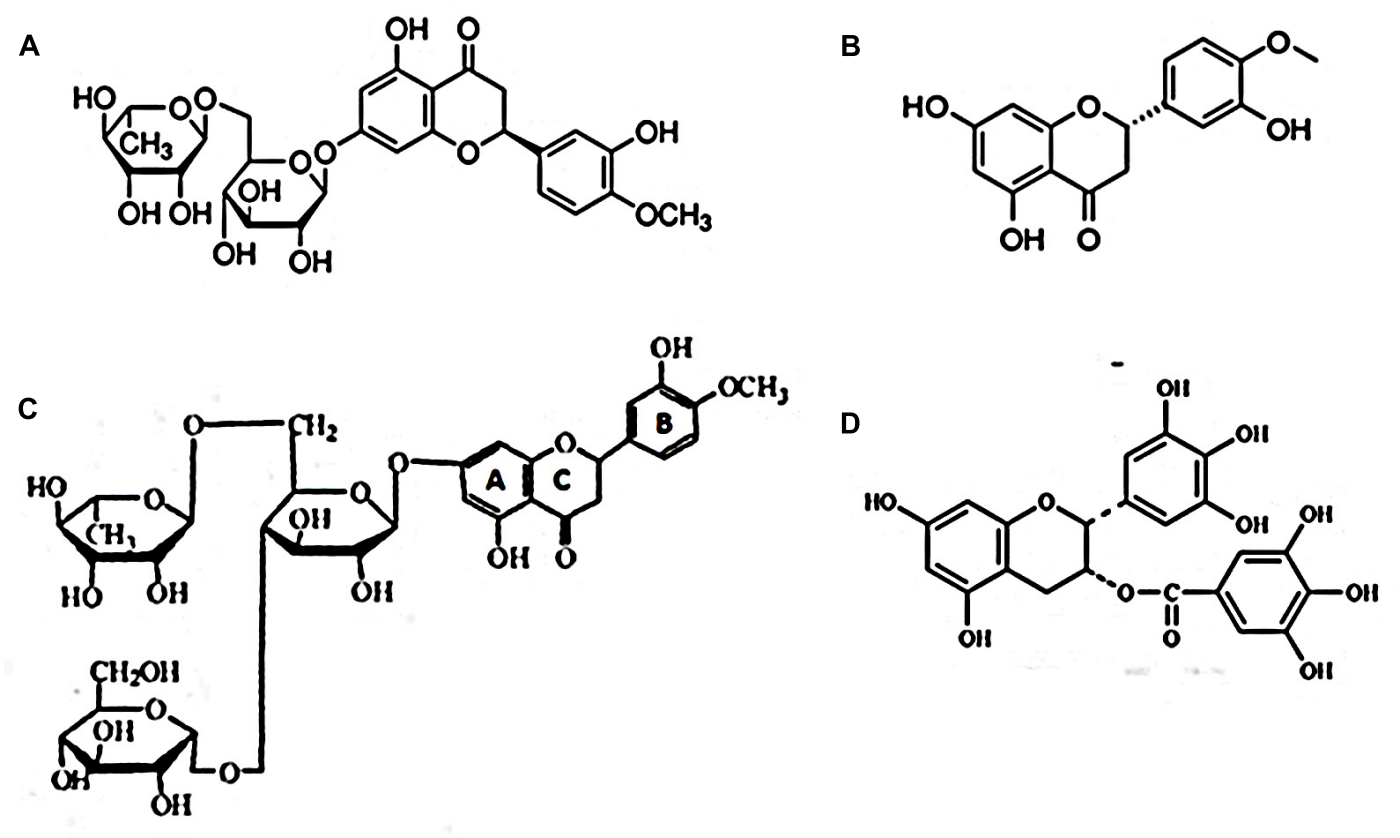
**Selected flavanones reported to possess antiviral activity. (A)** Hesperidin, a flavanone glycoside; **(B)** hesperitin, an aglycone of hesperidin; **(C)** α-glucosyl hesperidin (A semi-synthetic derivative of hesperidin. U.S. Patent number: 5,652,124); **(D)** EGCG.

Basic structural differences between naturally occurring flavonoids and the semisynthetic GH, as depicted on **Figure 7**, are suggested to be significant factors in one’s analysis of such natural plant products’ antiviral effects. The importance of flavonoid structural disparities were most clearly exemplified through comparative testing of four related catechin subgroups, wherein antiviral activity was shown to be related to the extent of molecule hydroxylation and the presence or absence of galloylation at the flavan-3-ol’s B and C rings, respectively, (Song et al., 2005; Gescher et al., 2011; Lipson et al., 2013). As alluded to above moreover, differences in antiviral activity to different viral species by the same flavonoid, occur as well. We suggest that no one secondary plant metabolite nor virus *per se*, may serve as an indicator or model system to the myriad of infectious agents circulating *ad perpetuity* throughout the world’s populations.

**FIGURE 8:**
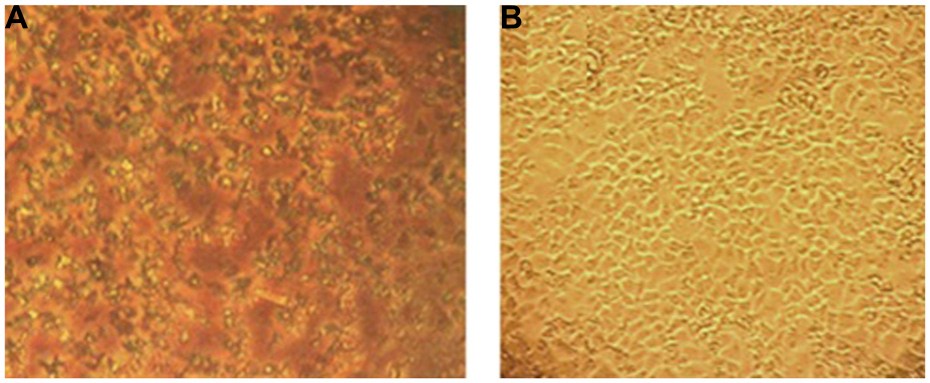
**Effect of EGCG on MA-104 cell cultures. (A)** Three hundred microgram EGCG induces a precipitation effect in microtitier wells containing MA-104 cell cultures maintaned in minimal essential medium plus supplements. **(B)** Untreated MA-104 monolayer culture. Amino acids and proteins are known to bond onto EGCG in a stacking pattern to which the above effect may be ascribed in (see text for details).

Increased concentrations of EGCG (ca., 300 μg/ml), observed under light microscopy, were observed to form particulate-like aggregates within cluster plate wells containing MA-104 cells maintained in culture medium (**Figure 8**). We ascribe this effect to an affinity by EGCG to constituent amino acids and perhaps low molecular weight proteins present in the system’s cell culture medium. EGCG is indeed known to bind to numerous amino acid and protein types (Ricardo-da-Silva et al., 1991; Charlton et al., 2002; Pascal et al., 2007). The addition of FBS to such a defined medium further adds to the plethora of amino acids and proteins in the pabulum used for cell growth and maintenance (Freshney, 2010). It is interesting to note that elevated concentrations of EGCG or GH failed to produce any cytotoxic effects in the current system (**Figure 6**). These findings probably reflect a binding of EGCG to amino acids or protein components present in the system’s culture medium, with MA-104 monolayers apparently refractory to potentially cytotoxic effects incurred by the flavonoid in question. A similar effect did not occur in our cell culture assay system using GH.

## Conclusion

Differences in GH and EGCG molecule structure appeared to significantly affect these flavonoid’s anti-RTV activity. Molecule solubility (obviating the need for potentially confounding organic carrier molecules) is important, but appears subordinate to flavonoid structure *per se.* Anti-RTV activity by EGCG was significantly more effective than GH, wherein the latter required inordinately high concentrations to affect a loss of viral infectivity to >1 log_10_. Cytotoxicity testing showed no loss of cellular integrity following monolayer treatment with either EGCG or GH. The precise mechanism(s) of ECGC or GH anti-RTV activity is ill defined. However, inasmuch as both EGCG and GH display amino acid/protein binding activity (Charlton et al., 2002; Jöbstl et al., 2004; Wang et al., 2007), one might suggest a binding of the virus in suspension by either flavonoid, affecting a blockage of capsid epitopes and in turn, reduced virus detection. Alternatively, one may not rule out a flavonoid-associated event taking place on the cellular level. Further studies are needed on the molecular level to identify whether EGCG, GH, or other flavonoid groups affect a down regulation of cellular protein synthesis which in turn, might compromise one or more stages in the RTV replication cycle.

## Conflict of Interest Statement

The authors declare that the research was conducted in the absence of any commercial or financial relationships that could be construed as a potential conflict of interest.
